# Changes in nitrogen availability lead to a reprogramming of pyruvate metabolism

**DOI:** 10.1186/s12870-018-1301-x

**Published:** 2018-05-04

**Authors:** Nazeer Fataftah, Christina Mohr, Mohammad-Reza Hajirezaei, Nicolaus von Wirén, Klaus Humbeck

**Affiliations:** 1 Institute of Biology/Plant Physiology department, Martin-Luther-University Halle-Wittenberg, Halle, (Saale) Germany; 2 Physiology and Cell Biology department, Leibniz Institute of Plant Genetics and Crop Plant Research (IPK), Gatersleben, OT Germany

**Keywords:** Barley, Nitrogen deficiency, Senescence, Transcriptomic, Metabolites

## Abstract

**Background:**

Low availability of nitrogen (N) severely affects plant growth at different levels, which can be reverted by the resupply of N. To unravel the critical steps in primary metabolism underlying the growth adjustment in response to changes in N availability, transcriptomic and comprehensive metabolite analyses were performed in barley using primary leaves at early and later stages of N deprivation, and after N resupply to N-deficient plants.

**Result:**

N deficiency in leaves caused differential regulation of 1947 genes, mostly belonging to the functional classes photosynthesis, cell wall degradation, lipid degradation, amino acid degradation, transcription factors, phytohormone metabolism and receptor-like kinases. Interestingly, 62% of the genes responding to low N were regulated in the opposite direction after two days of N resupply. Reprogramming of gene transcription was linked to metabolic rearrangements and affected the metabolism of amino acids and sugars. The levels of major amino acids, including Glu, Asp, Ser, Gln, Gly, Thr, Ala, and Val, decreased during primary leaf age and, more pronounced, during low N-induced senescence, which was efficiently reverted after resupply of N. A significant decrease was observed for pyruvate and metabolites involved in the TCA cycle under low N, and this was reverted to initial levels after 5 days of N resupply. Correspondingly, transcript levels of genes coding for pyruvate kinase, pyruvate dehydrogenase, and pyruvate orthophosphate dikinase followed the same trend as related metabolites.

**Conclusion:**

Our results show that upon N limitation a specific pathway for remobilization at the link between glycolysis and TCA cycle in barley is established that is at least partly regulated by a strict reprogramming of the gene coding for pyruvate orthophosphate dikinase. Further analysis of this pathway, its regulatory levels and biochemical changing of pyruvate metabolism enzymes in response to N availability is needed to determine the link between N status and primary metabolism.

**Electronic supplementary material:**

The online version of this article (10.1186/s12870-018-1301-x) contains supplementary material, which is available to authorized users.

## Background

Nitrogen (N) is a macronutrient for plants and often a limiting factor for their growth and development. Plants obtain N as nitrate and ammonium from the soil, with urea or amino acids making a minor contribution [1]. It was estimated that 85–90 million metric tons of nitrogenous fertilizers are added annually to soils worldwide [2], and N fertilization represents a major expense in plant production. Furthermore, incomplete capture and conversion of nitrogen fertilizers into leachable or diffusible N forms cause soil and water pollution, as well as global warming. Lowering fertilizer input and breeding plants with higher nitrogen use efficiency (NUE) is one of the main goals of research on plant nutrition [3]. To approach this goal, a better understanding of how crop plants reprogram their cellular functions at different regulatory levels in response to alterations in N supply is needed. After N is taken up by plant roots, it is assimilated in roots and transferred to the shoot in the form of amino acids or of inorganic nitrate and ammonium. When nitrate reaches the shoot, the first step of N assimilation is to convert nitrate to nitrite, which is catalyzed in the cytosol by nitrate reductase (NR) [4]. Then, nitrite is translocated to the chloroplast where it is reduced to ammonium by nitrite reductase (NiR), followed by assimilation of ammonium into glutamate and glutamine by the Gln synthetase/Glu synthase (GS/GOGAT) cycle [5].

When N supply is limited, cellular functions change in a way that precious N resources are sustainably recycled and utilized to survive under these adverse conditions. For example, a contingent of rubisco is degraded to liberate N, photosynthesis is down-regulated, peptidases are induced to remobilize protein-N, cytosolic ammonium assimilation into glutamine is promoted and high-affinity transporters for re-translocation of nitrogenous compounds are upregulated, mainly in source leaves [6–9]. In addition, low N availability causes premature senescence and thereby enhances translocation of N to sink tissues. However, resupply of N after a period of shortage can stop this metabolic programming [10, 11], indicating N-sensitive regulatory pathways efficiently and coordinately controlling growth. While previous investigations addressed changes either in the transcriptome or in the metabolome mostly during N shortage [6, 11–13], the present study aimed at elucidating the regulatory mechanisms in leaves in response to low N, and to N resupply by combining gene transcription and metabolite analysis. For these studies, we used barley as a model cereal, not only because of its economic importance (it ranks fourth among cereals worldwide) [14] but also because its genome sequence, EST collections, molecular markers, DNA arrays and other omics-derived data are available [14].

## Methods

### Plant culture and sampling

Barley seeds (*Hordeum vulgare* L. cv Golden Promise from gene bank of IPK Gatersleben, Germany, Accession number HOR 16645) were germinated and grown in 0.5× nutrient solution. Eight days after germination (DAG) the seedlings were transferred to 5 l pots supplied with full nutrient solution containing 2 mM Ca(NO_3_)_2_, 0.5 mM K_2_SO_4_, 0.5 mM MgSO_4_, 0.1 mM KCl, 0.1 mM KH_2_PO_4_, 1.0 μM H_3_BO_3_, 2.5 μM MnSO_4_, 0.5 μM ZnSO_4_, 0.2 μM CuSO_4_, 0.01 μM (NH_4_)_6_Mo_7_O_24_, and 100 μM Fe(III)-EDTA. This treatment was also used as control treatment. Another group of plants was subjected to N deficiency by substituting 2 mM Ca(NO_3_)_2_ by 0.06 mM Ca(NO_3_)_2_ and 1.94 mM CaCl_2_ for ionic balance. The N-deficient plants were grown for 15 d under N deficiency. After 10 d (at 18 DAG) of N deficiency treatment, a group of deficient plants was resupplied by 2 mM Ca(NO_3_)_2_. The hydroponic system was permanently aerated and maintained in a controlled-environment chamber at 70% humidity, 18 °C/8 h and 20 °C/16 h dark/light cycle at 210 μmol photons m^− 2^ s^− 1^. The nutrient solution was changed every three days (2 d before harvesting). The primary leaves were harvested (after 8 h of light) in three-day intervals, and stored at − 80 °C for further analyses.

### Chlorophyll content and root: Shoot ratio

Relative chlorophyll content was determined using a SPAD (Soil Plant Analysis Development) analyser (Minolta, by Hydro Agri, Dülmen, Germany), which measures the transmission of wavelengths 650 and 940 nm (measuring area = 6 mm^2^) absorbed by chlorophyll in intact leaves. Each data point represents the mean value of 10 independent measurements. For the calculation of root: shoot ratio, the plant material was dried at 70 °C for two days and weighted.

### RNA isolation

At each defined time point, ca. 0.5 g of frozen, homogenized primary leaf material was used for RNA preparation. Total RNA was isolated from leaves with a TRIzol-based method according to Chomczynski and Mackey [15] and quantified by NanoDrop-spectrophotometer (NanoDRop Technologies Inc., USA). TRIzol reagent (38% phenol, 0.8 M guanidinium thiocyanate, 0.4 M ammonium thiocyanate, 0.1 M sodium acetate, pH 5, 5% glycerol) was used. To verify the quality of RNA, 1 μg of total RNA was fractionated on a 1% (*w*/*v*) TAE agarose gel, stained with ethidium bromide and visualized under UV light.

### Quantitative RT-PCR (qRT-PCR)

Total RNA was isolated as described in the section above and treated with RNase-free DNaseI (MBI Fermentas, St Leon-Rot, Germany). One microgram of total RNA was reverse transcribed with Superscript III reverse transcriptase (Invitrogen, Karlsruhe, Germany) in a volume of 20 μl to generate first-strand cDNA, according to the supplier’s instructions. PCR was performed in an iCycler (BioRad, Munich, Germany) in a total volume of 15 μl, including cDNA, corresponding to 6 ng starting total RNA, 1X Platinium® SYBR® Green qPCR SuperMix-UDG (Invitrogen), 0.3 μM of each gene-specific primer and 10 μM fluorescein (BioRad) as passive reference dye for well factor calibration. The following PCR program was used: 2 min incubation at 50 °C, 1 cycle at 95 °C for 2 min to activate the included HotStart-Taq-polymerase, followed by 40 cycles at 95 °C for 15 s, 58 °C for 15 s and extension phase at 60 °C for 15 s. Subsequent to the normal PCR, determination of a melt curve of the amplified PCR products was carried out. cDNA was checked by the expression of actin and PP2A genes. The relative expression rate of genes of interest in senescing leaves and leaves after N re-supply treatment relative to the mature controls at 11 DAG was calculated. Each data point is based on 3–7 biological replicates. Data were normalized to the reference gene actin. The primer list is provided in Additional file 1 Table S1.

### Microarray analysis

Total RNA was isolated as described in the section above and treated with RNase-free DNaseI (MBI Fermentas, St Leon-Rot, Germany). Three biological replicates were carried out. The biological replicate was a pool of ten primary leaves. RNA integrity was confirmed using the Bioanalyser system (Agilent Technologies). For cRNA synthesis, 100 ng RNA was used following Cy3-labelling with a Low Input Quick Amp Labelling Kit (Agilent Technologies). Labeling efficiency, amount and quality of cRNA were assured using an ND-1000 Spectrophotometer (NanoDrop Technologies, Wilmington, USA) and Bioanalyser system. For fragmentation and array loading (Gene Expression Hybridization Kit, Agilent Technologies), 600 ng labeled cRNA was used. Hybridization was performed with the array that has been designed as described in Kohl et al. [6]. Hybridization was done for 17 h at 65 °C. After washing (Gene Expression Wash Buffer Kit, Agilent Technologies) and drying, arrays were scanned at 5 μm resolution using an Agilent Technologies Scanner G2505C. Resulting images were evaluated (determination of spot intensities, background correction) with Feature Extraction V11.5 (Agilent Technologies).

Data evaluation was done with Genespring V12.5 (Agilent Technologies). Values were log_2_ transformed and quantile normalized before relative expression values were calculated by subtracting the median expression of each probe from the other values of this specific probe (baseline transformation). Ratios of gene expression levels were calculated (log_2_-fold change (FC)). Expression levels in N-deficient plants were compared to those in control plants at the same time points (17, 20 DAG), and in plants resupplied with N to those in plants further kept under low N conditions at the corresponding time point (20 DAG). After removing outliers and transcripts without significant expression at any time point, ANOVA (*P* ≤ 0.05, FC ≥ 2) and FDR correction (Benjamini-Hochberg) was performed as described in Kohl et al. [6].

### Determination of Glc, Frc, Suc, starch, and amino acids

For these analyses, 50 mg frozen leaf material was homogenized in liquid nitrogen, dissolved in 0.75 ml of 80% (*v*/v) ethanol and incubated at 80 °C for 60 min. Crude extracts were centrifuged at 14,000 rpm at 4 °C for 5 min and the upper phase was concentrated in a speed vacuum concentrator (Christ, RVC 2–33 IR, Germany) at 45 °C for 180 min. The pellet was re-suspended in 0.3 ml HPLC-grade water and shaken for 15 min at 4 °C for the measurement. The remaining insoluble material was kept for starch measurements. Soluble sugars (including Glc, Frc, and Suc) and starch were determined in primary leaves according to Ahkami et al. [16], and Hajirezaei et al. [17].

To detect amino acids, a fluorescing reagent AQC (6-aminoquinolyl-N-hydroxysuccinimidylcarbamate) was used. AQC was dissolved in 3 mg ml^− 1^ of acetonitrile and incubated at 55 °C for 10 min. Twenty μl of the extracts were derivatized in a cocktail containing 20 μl of the fluorescing reagent ACQ, 160 μl of a 0.2 M boric acid buffer (pH 8.8) in a final volume of 200 μl. The solution was incubated at 55 °C for 10 min. The separation of derivatized samples was carried out with a reversed phase HPLC system (Waters, Germany) consisting of a gradient pump (Alliance 2795 HT, Waters, Germany), a degassing module, an autosampler and a fluorescence detector (Waters 2475, Germany). A reversed phase column (XBridge; 150 mm, 5 μm) was used for separation and detection of amino acids at an excitation wavelength of 300 nm and an emission wavelength of 400 nm. The gradient was accomplished with buffer A containing 140 mM sodium acetate, pH 5.8 (Suprapur, Merck) and 7 mM triethanolamine (Sigma, Germany). Acetonitrile (Roti C Solv HPLC, Roth) and purest HPLC water (Geyer, Germany) were used as eluents B and C. Twenty stable amino acid standards purchased from Sigma-Aldrich, Germany, at serial dilutions were used to generate the standard curves for targeted quantitative analysis of the corresponding amino acid. Chromatograms were recorded using the software program Empower Pro. To determine the absolute concentrations, the extracted amount of metabolites from the Empower Pro software was normalized to the extraction volume and started tissue material.

### Determination of nitrate

The same extracts that are used for carbohydrate and amino acids analyses were also analysed for nitrate using an ion chromatography system connected to a conductivity detector (Dionex, Thermofisher Germany). The control of the complete system, recording of the spectra and data acquisition was performed with the Chromeleon software, release 7.0 (Dionex GmbH, Germany). To separate anions, an ICS5000 system (Dionex, Germany) was used including a gradient pump DC, an autosampler AS-AP and a conductivity detector. Separation of the anions was carried out using a high capacity ion exchange column (AS11-HC, 250 × 2 mm) connected to a guard column of the same material (AG 11-HC, 10 × 2 mm) and an ATC-1 anion trap column, which is placed between the eluents and separation columns to remove the anions present in the solutions. The gradient was accomplished with the purest water (buffer A, Millipore) and a concentrated potassium solution EGCIII KOH (Dionex, Germany, buffer B), and the corresponding gradient was produced using an eluent generator EG-SP (Dionex Germany). The column was equilibrated with a mixture of buffer A (96%) and buffer B (4%) at a flow rate of 0.32 ml per minute and heated at 35 °C during the whole measurement. The gradient was produced in 25 min run by changes of the buffer B as follows: 0–4 min at 4%, 4–10 min at 15%, 10–18 min at 80% and 18–25 min at 4%.

### Determination of phosphorylated sugars, glycolytic and TCA cycle metabolites

To determine the phosphorylated sugars, glycolytic and TCA cycle metabolites, 100 mg of finely powdered material was extracted using 1 ml (*v*/v) (1:1) ice-cold methanol and chloroform. Subsequently, 0.3 ml of LC-MS water was added to each tube. The sample was mixed and kept on ice for 20 min. The mixtures were then centrifuged for 10 min at 14,000 rpm and 4 °C. Thereafter, the upper phase containing methanol/water was transferred to new Eppendorf tubes and concentrated at 40 °C for 2 h in a speed vacuum concentrator (Christ, RVC 2–33 IR, Germany). The remaining pellet was re-suspended in 0.3 ml of LC-MS water and was stored at − 80 °C for metabolite analysis.

For quantification of extracted metabolites, a targeted metabolite analysis was performed using external standards. 0.1 ml of sample volume extracted in the section above was filtered at 2000 g for 90 min using a multiscreen filter plate (multiscreen ultracel-10 ultra-filtration membrane 10,000 NMWL). The IC-MS-MS instrumentation consisted of a Dionex ICS5000 (Dionex, Idstein, Germany) coupled to a triple Quad MS-MS (6490, Agilent, Germany). Metabolites were separated on the same column as described for anions in the section above except that quantitative analysis was carried out using an Agilent 6490 triple quadruple mass spectrometer (Agilent, Germany). Electron spray ionization (ESI)-MS/MS was set as follows: gas temperature 350 °C, drying gas flow rate 12 L min^− 1^, nebulizer pressure 35 psi, capillary voltage ±3.5 kV. The fragmentor voltage and collision energy were optimized for each compound individually by tuning standards with a defined concentration. Primary metabolites were detected in the negative ion mode using multiple reactions monitoring (MRM). ^13^C-pyruvate was added to each sample as internal standard before analysis. Thirty stable standards (Sigma-Aldrich, Germany) at serial dilutions were used to generate the standard curves for targeted quantitative analysis of the corresponding metabolites. Metabolome data processing including peak detection and retention time alignment was carried out and the data were extracted using the MassHunter software version B.03.01 (Agilent Technologies, Germany). Quantification of metabolites was performed by creating a batch for each sample sets using the Quantitative Analysis (QQQ) software (Agilent Germany). The QQQ software serves as a tool for absolute quantitation by linking the target metabolite to its standard reference and the corresponding m/z ratio as well as it uses the reference calibration curve to automatically determine the amount of the corresponding metabolite. Then to determine the absolute concentration, the extracted amount of metabolites from the software were normalized to the extraction volume and started tissue material.

### Statistical analyses

The two-way ANOVA analysis was performed by InfoStat/Student program [18] and LSD according to Fisher were calculated for statistical analyses. A difference at *P* ≤ 0.05 was considered as significant.

## Results

### Leaf development under different N regimes

**Fig. 1:**
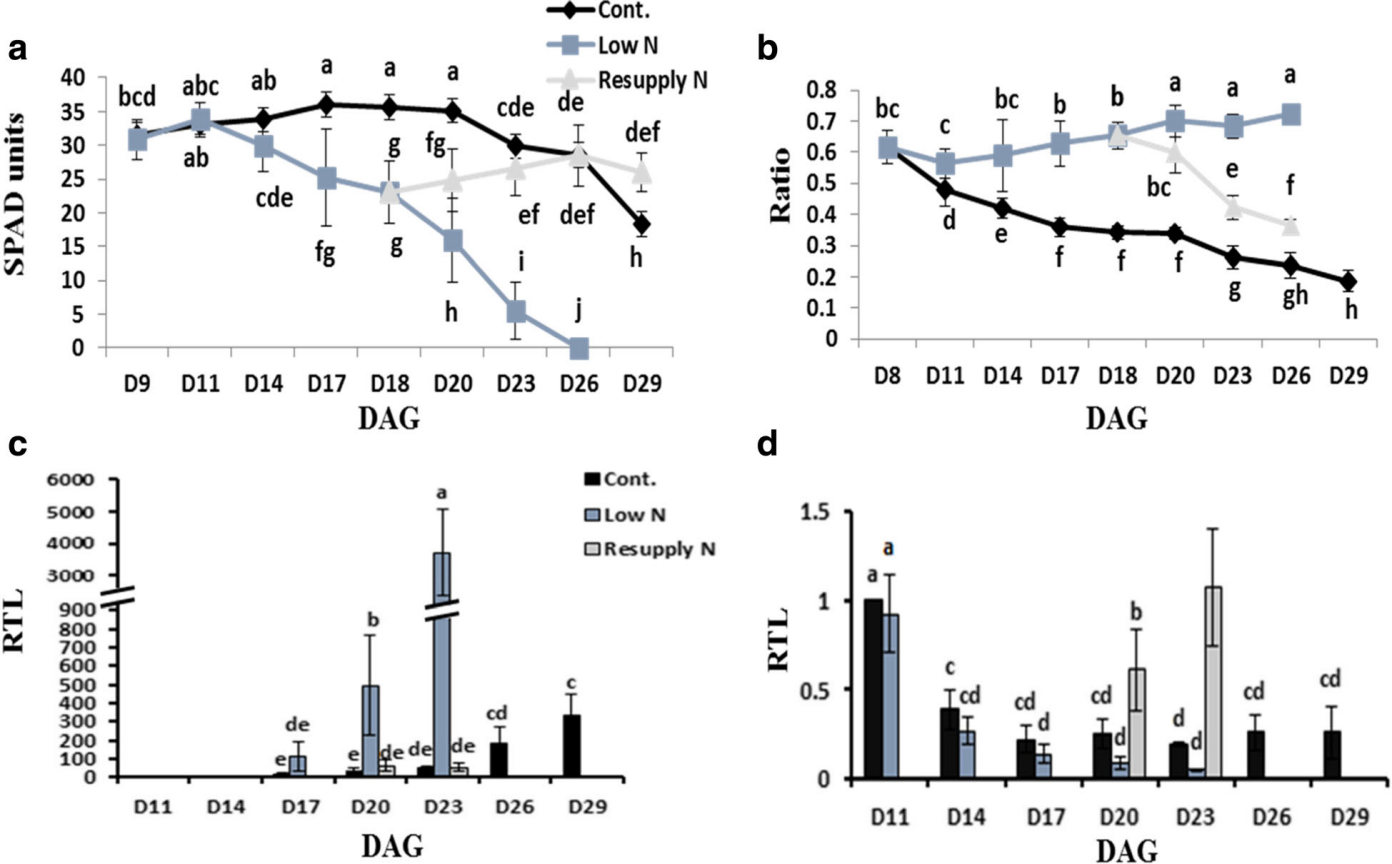
Age-dependent progression of leaf senescence under different N regimes. **a** Relative chlorophyll content of primary leaves as determined by SPAD measurements. **b** Root/shoot dry biomass ratio. **c** Relative transcript levels (RTL) of *S40* and **d** of *GS2*. For C and D, actin was used as a reference gene. All transcript levels were normalized to those of control plants at 11 DAG. DAG: days after germination. Data represent means ±SD (*n* ≥ 10 plants for A and B; *n* = 3–5 for C and D). Different letters represent significant differences according to two-way ANOVA at *p* ≤ 0.05

To investigate the impact of N availability on the development of primary leaves, barley plants were grown under three N regimes, control (2 mM Ca(NO_3_)_2_), continuous N starvation (0.06 mM Ca(NO_3_)_2_) or N resupply (2 mM Ca(NO_3_)_2_) after a period of N starvation for 10 d (corresponding 18 days after germination (DAG)). Chlorophyll content, as a marker for the developmental stage of leaves, stayed high in primary leaves of control plants up to 20 DAG. Thereafter, it started to decrease slightly reflecting the onset of senescence, which is the last step of leaf development (Fig. 1a). In consequence of N shortage, chlorosis started already at 14 DAG and chlorophyll content severely decreased compared to control plants (Fig. 1a). Notably, when N was resupplied to N-starved plants, chlorophyll content recovered and reached the levels of control plants (Fig. 1a). This indicated that resupply of N to senescing leaves prevented further progression of N deficiency-induced leaf senescence. In addition, N deficiency inhibited shoot growth, as reflected by an increase in a root-to-shoot ratio that reverted after N resupply (Fig. 1b).

To analyze the effect of N nutritional status at the molecular level, and to document the developmental switch from a mature, photosynthetically active growth to senescence, transcript levels of the senescence-associated gene *S40* [19] and of the N-metabolism gene glutamine synthetase 2 (*GS2*) [10] were determined via qPCR and normalized to those of control plants at 11 DAG. In control plants, transcript levels of *S40* increased at later stages when developmental senescence had set in primary leaves and reached their maximum at 29 DAG. Under N deficiency, *S40* transcript levels increased earlier and reached much higher levels compared to those in control plants at the same time points (Fig. 1c). Interestingly, resupply of N to N-starved plants clearly suppressed the increase in *S40* transcript levels (Fig. 1c). By contrast, *GS2* transcript levels ceased with leaf age in control plants, and this was even more pronounced under low N conditions (Fig. 1d). Notably, N resupply to N-starved plants strongly induced transcript levels of *GS2* in primary leaves, even to much higher levels than those in control plants of the same age (Fig. 1d).

### Transcriptome analysis of primary leaves during N deprivation and after resupply of N

A comparative transcriptome profiling was performed in primary leaves under control and low N at 17 and 20 DAG, As well as N resupply conditions at 20 DAG. Gene transcription levels in N-deficient plants were compared to those in control plants at the same time points (17, 20 DAG), and in plants resupplied with N to those in plants further kept under low N conditions at 20 DAG. Data analysis revealed that under N deficiency 751 and 895 transcripts were induced, and 864 and 1053 transcripts were down-regulated at least by twofold at 17 DAG or 20 DAG, respectively (Additional file 2 Table S2). *S40*, a senescence marker gene (Fig. 1c), was also found in microarray data to be up-regulated or down-regulated in response to N deficiency or resupply of N, respectively. The data also revealed that 4083 transcripts were differentially regulated by N resupply (at 20 DAG), with 2220 transcripts being up-regulated and 1863 down-regulated (Additional file 2 Table S2). Of 1948 transcripts differentially regulated at 20 DAG of N deficiency, 1208 transcripts (62%) reversed their expression changes after 2 d of N resupply (20 DAG), therefrom 665 being again upregulated and 543 being again downregulated.

**Fig. 2:**
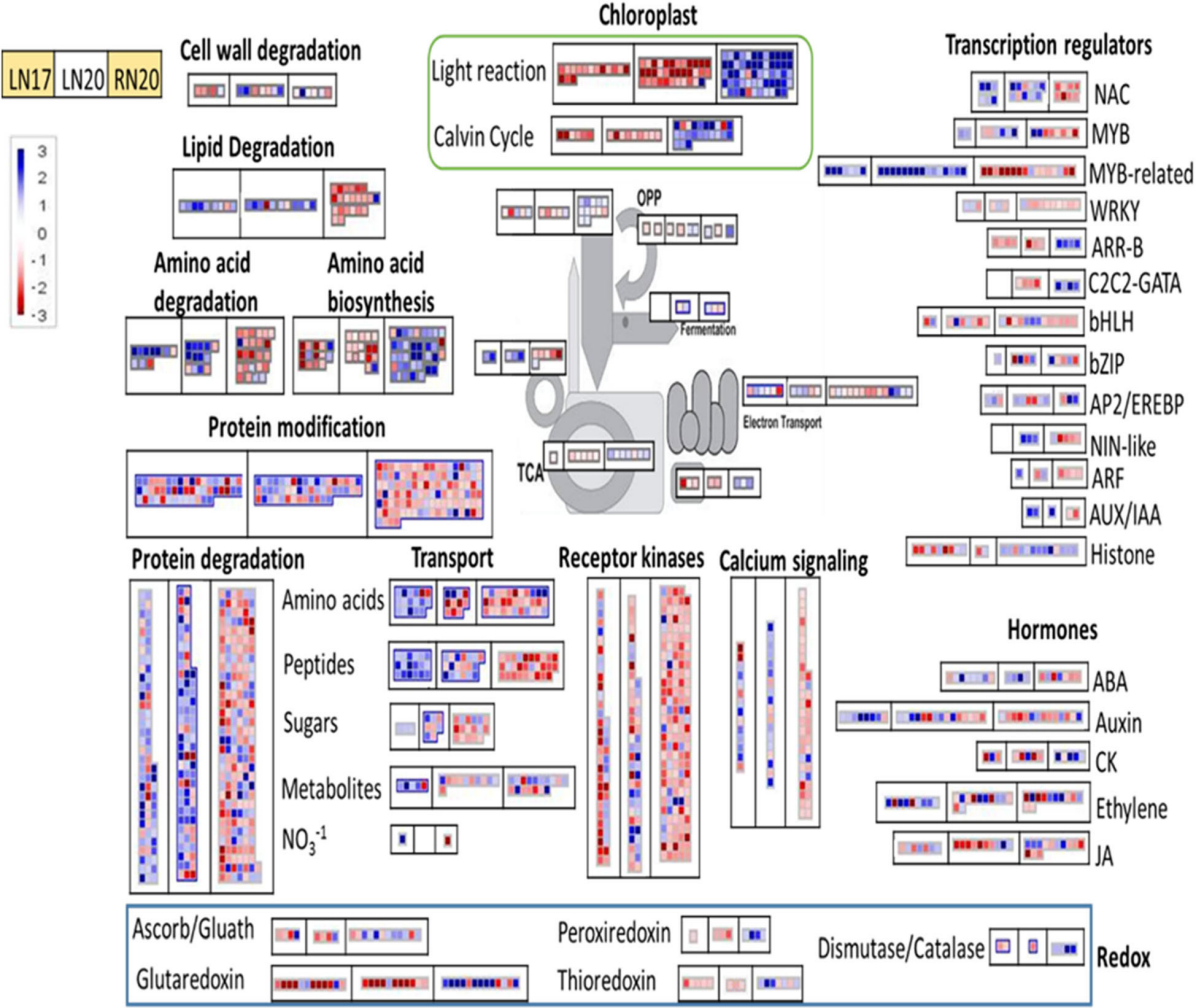
Functional classification of N-responsive genes by Mapman software. Small squares in boxes represent individual transcripts belonging to different functional categories. Each category consists of three boxes, where the left box shows differentially regulated genes in LN17 (low N at 17 DAG compared to control at 17 DAG), the middle box represents differentially regulated genes in LN20 (low N at 20 DAG compared to control at 20 DAG), and the right box represents differentially regulated genes in RN20 (resupply of N at 20 DAG compared to low N at 20 DAG). Blue color indicates up-regulation and red color down-regulation of gene transcription. DAG: days after germination

Further analysis using Mapman software [20] sorted differentially regulated genes into functional classes. About 63% of all differentially regulated genes could at least be assigned to one functional class. A number of functional subcategories that were selected from the MapMan annotation are visualized in Fig. 2. Transcripts involved in photosynthesis (light reaction and Calvin cycle) were clearly down-regulated in response to N shortage, reflecting the degradation of chloroplasts during low N-induced senescence. When N was resupplied, these genes related to chloroplast function were mostly up-regulated again. Genes involved in catabolic processes and belonging to the subcategories cell wall degradation, lipid degradation, amino acid degradation or protein degradation were mostly upregulated during N deficiency at both time points. In addition, many genes involved in protein modifications were also upregulated under N shortage. In general, the regulation of most of these genes during N shortage was reverted when N was resupplied. On the other hand, genes involved in amino acid biosynthesis were mostly down-regulated during N-deprivation and induced when N was resupplied after N starvation. Senescence is characterized by efficient translocation of organic compounds and mineral elements from senescing leaves to other growing parts of the plant. Accordingly, several transcripts encoding transporters for amino acids, peptides, and sugars were up-regulated during N deficiency-induced senescence. This induction was more obvious at 17 DAG (Fig. 2). Many genes coding for transcription factors, phytohormone metabolism, and signaling, as well as genes coding for receptor-like kinases, calcium signaling, and redox-regulation responded to N. Especially, NAC-type, MYB, and MYB-related type transcription factors were clearly up-regulated in response to N deficiency. Moreover, these transcription factors were down-regulated upon resupply of N. Other transcription factors showed an opposite regulation, i.e. they were down-regulated in N-deficient plants, but up-regulated upon resupply of N. This includes a B-type response regulator (ARRs-B) and a zinc finger domain C2C2-GATA transcription factor. Interestingly, many genes involved in calcium signaling also showed a significant up-regulation during N deprivation, and a substantial downregulation, when N was re-supplied, indicating reprogramming of the calcium signaling machinery in response to N availability. Genes encoding other regulatory factors, such as receptor kinases and hormones, showed no uniform response of their functional category, indicating specific functions of the different receptor kinases and hormonal pathways during low N-induced senescence.

### Changes of soluble amino acids in response to N supply

**Fig. 3:**
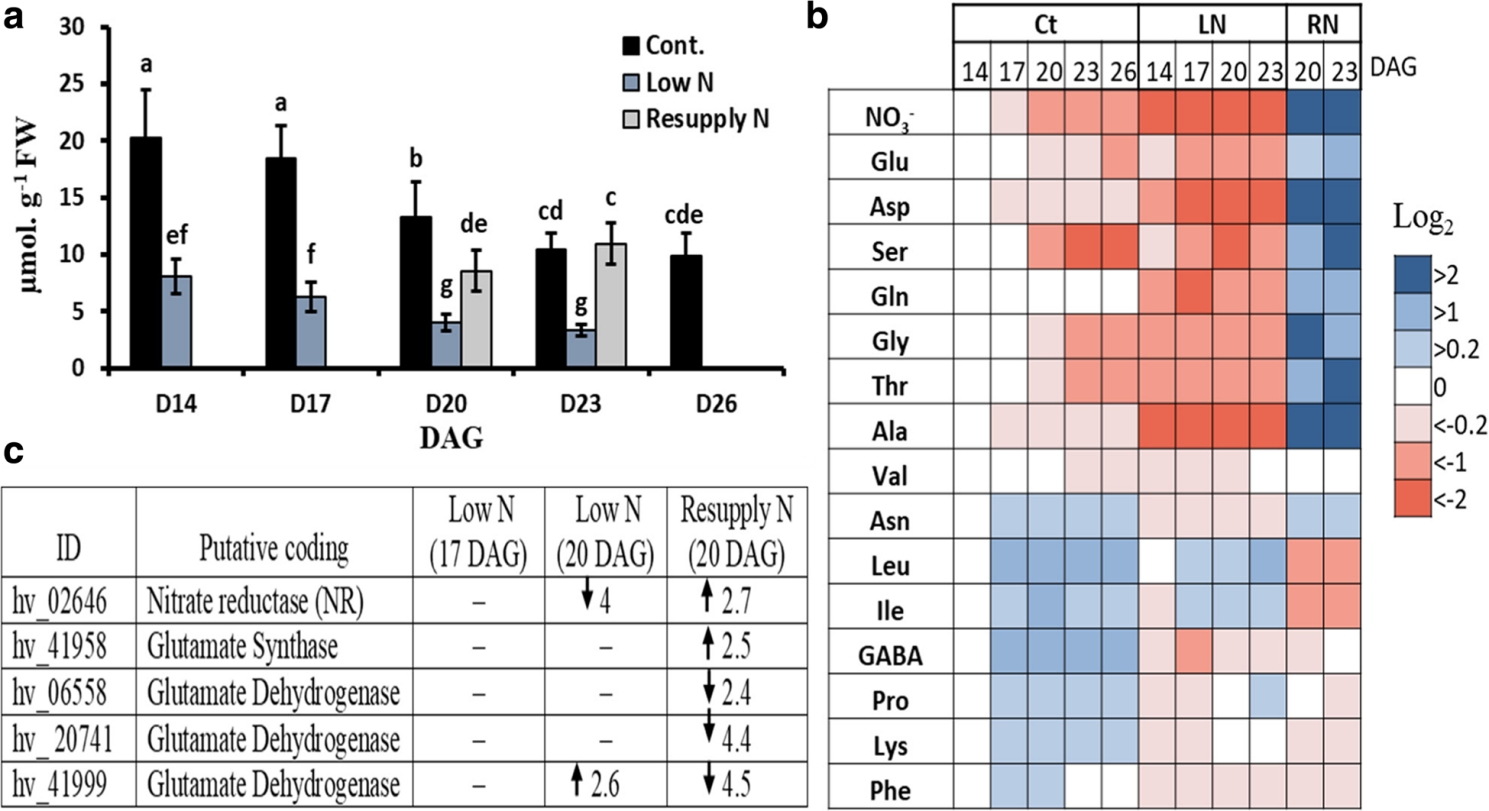
Amino acid profiling in leaves in response to N supply. **a** The total concentration of measured amino acids. **b** Heat-map of relative concentrations of individual amino acids; Results of control plants were logarithmic (log_2_) normalized to the values of control plants at 14 DAG; Results of low-N plants were logarithmic (log_2_) normalized to those of control values at the same time point, while results of N resupply plants were logarithmic (log_2_) normalized to those of low N values at the same time point. **c** Changes in transcript levels of differentially regulated genes putatively related to primary N metabolism that were determined via microarray analysis; the values represent fold-changes. Up arrow: Upregulated; down arrow: downregulated. Ct: control; LN: low N; RN: resupply of N. Bars indicate means ± SD (*n* = 5–9). Different letters represent significant differences according to two-way ANOVA at *p* ≤ 0.05

At 14 DAG, the total concentration of measured amino acids (sum of 15 detected AAs) was 20 μmol g^− 1^ FW and then continuously decreased with leaf age in control plants down to 10 μmol g^− 1^ FW at 26 DAG (Fig. 3a). As expected, the total concentration of amino acids was significantly lower in primary leaves of N-deficient plants (Fig. 3a). Significant differences between control and N-deficient plants were already detected at 11 DAG (data not shown). After 5 d of N resupply (at 23 DAG), the total amino acid concentration recovered to the level of control plants. Levels of NO_3_^−^ and major individual free amino acids (Glu, Asp, Ser, Gly, Thr, Ala and Val) followed the same pattern as total amino acids, with depletion during developmental senescence, a stronger decrease in low N and accumulation after resupply of N, thus reflecting major alterations in amino acid metabolism during leaf development in response to N availability. The level of Gln, the major transported amino acid out of senescing leaves, remained unchanged and thus in contrast to other amino acids during developmental senescence. A sub-group of amino acids (Asn, Leu, Ile, GABA, Pro, Lys, and Phe) showed a different response, with increasing levels during developmental senescence (Fig. 3b). In contrast to other amino acids, relative concentrations of Leu and Ile and at day 23 Pro even increased at low N and substantially decreased after N resupply (Fig. 3b).

These major changes in amino acid composition during adaptation to the N regime were reflected by changes in the mRNA levels of corresponding genes. Our array results confirmed a strong down-regulation of the expression of nitrate reductase (hv_02646), which governs the flux of nitrate into the assimilation pathway (Fig. 3c). Upon resupply of N, this down-regulation was reversed. A similar up-regulation in response to increased N availability after N starvation was observed for glutamate synthase (hv_41958), playing a primary role in ammonium incorporation into glutamine and glutamate (Fig. 3C). Transcript levels of three glutamate dehydrogenase genes (hv_06558, hv_20741, and hv_41999) exhibited an opposite behavior, with down regulation after resupplying of N. While the N status modulates a group of transcripts related to the metabolism of certain amino acids, only a few changes in amino acid contents were related to transcriptional changes.

### Changes in sugar concentrations in response to N availability

**Fig. 4:**
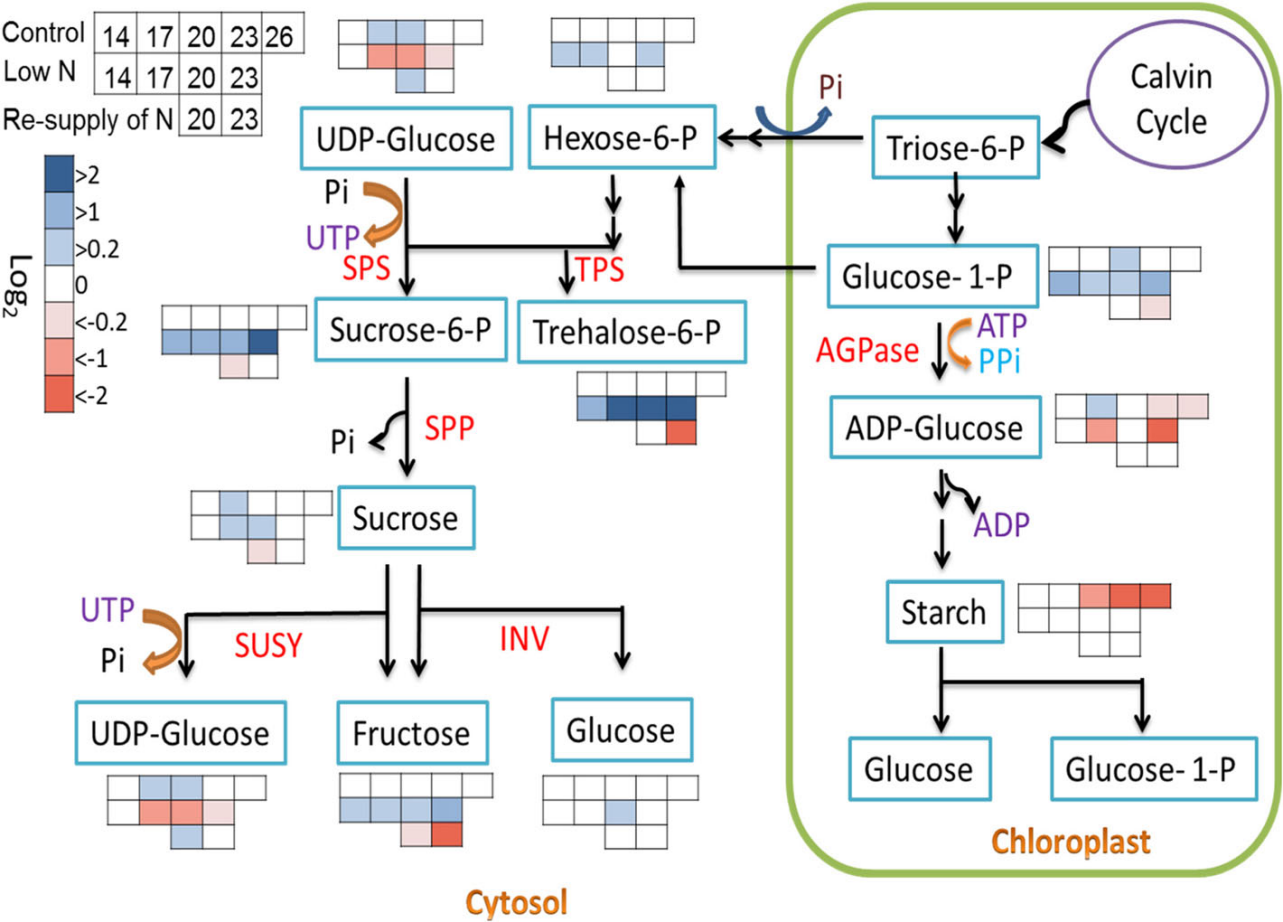
Sugar metabolism in response to the N status. Concentrations of different sugars in control plants were logarithmic (log_2_) normalized to the values of control plants at 14 DAG; Values from low-N plants were logarithmic (log_2_) normalized to those of control values at the same time point, while values of N resupplied plants were logarithmic (log_2_) normalized to those of low-N plants at the same time point. (*n* = 5–9); *P* ≤ 0.05; AGPase: ADP-Glc pyrophosphorylase; TPS: trehalose-6-P synthase; SPS: sucrose-phosphate synthase; SPP: sucrose-phosphate phosphatase; SUSY: sucrose synthase; INV: invertase

Nitrogen and carbon metabolism are closely connected since carbon forms the skeleton for N-containing metabolites. Targeted metabolite profiling revealed that hexose-6-P, sucrose-6-P, trehalose-6-P, sucrose, UDP-glucose, glucose and fructose concentrations were rather stable in control plants (Fig. 4). In contrast, the starch content significantly dropped during developmental senescence. Low N treatment caused a more differentiated response to sugar levels. While sucrose-6-P, trehalose-6-P and also fructose and glucose-1-P showed increased levels when compared to control plants, UDP-glucose and ADP-glucose levels were lower in low N-treated plants (Fig. 4). Resupply of N to N-starved plants resulted in a significant decrease in trehalose-6-P and fructose, mainly at 23 DAG (Fig. 4).

Transcript profiling showed that many genes related to sucrose metabolism including sucrose synthase and cell wall invertase were up-regulated in N-deficient plants, while N resupply suppressed these genes. Interestingly, the genes coding for starch-degrading enzymes were mainly up-regulated after 2 d of N resupply (Table 1).

**Table 1:** Differentially regulated genes putatively related to sugar metabolism as determined via microarray analysis; the values represent fold-changes. Up arrow: Upregulated; down arrow: downregulated. Ct: control; LN: low N; RN: resupply of N

ID	Putative coding	Low N (17 DAG)	Low N (20 DAG)	Resupply of N (20 DAG)
hv_03530	Sucrose synthase (SuSy)	↑9.1	↑9.1	↓7.5
hv_03531	Sucrose synthase (SuSy)	↑8.7	↑8.5	↓8.1
hv_20661	Sucrose synthase (SuSy)	–	–	↓4.5
hv_19372	Cell wall invertase	–	2.6	–
hv_22536	Cell wall invertase	–	–	↓13.5
hv_03914	Vacuolar invertase	–	–	↓60.5
hv_42447	Vacuolar invertase	–	–	↓25.3
hv_23265	Fructokinase	↓15.1	↓5.5	↑27.9
hv_11681	Fructokinase	–	–	2.3
hv_12878	Starch cleavage-beta amylase	–	–	−2.5
hv_22195	Starch cleavage-beta amylase	–	↓2.2	↑3
hv_01022	Degradation starch.D enzyme	–	–	↑2.5
hv_02206	Degradation starch.D enzyme	–	–	↑4.7
hv_02207	Degradation starch.D enzyme	↓3.5	–	↑3.5
hv_02208	Degradation starch.D enzyme	–	–	↑4.6
hv_04176	Degradation starch.D enzyme	–	↓4.1	↑3.7
hv_45799	Degradation starch.transporter	–	↓2.5	↑3.7
hv_08578	Glucan water dikinase	↓4.8		–
hv_03439	Degradation starch.ISA3	–	↓2.1	–
hv_36596	Starch synthase	–	–	2.5
hv_05868	Starch branching enzyme	–	–	↑43.4
hv_23288	Starch debranching enzyme	–	↓4.6	↑4.7
hv_12166	T6P synthase (TPS)	–	↓2.1	↑2.2
hv_10051	T6P phosphatase (TPP)	↓2.9	–	–
hv_41351	T6P phosphatase (TPP)	–	–	↓3
hv_04008	Potential TPS/TPP	–	↓2.7	–
hv_04009	Potential TPS/TPP	–	↓5.6	↑6.3

### N-responsive metabolic rearrangement at the link between glycolysis and TCA cycle

**Fig. 5:**
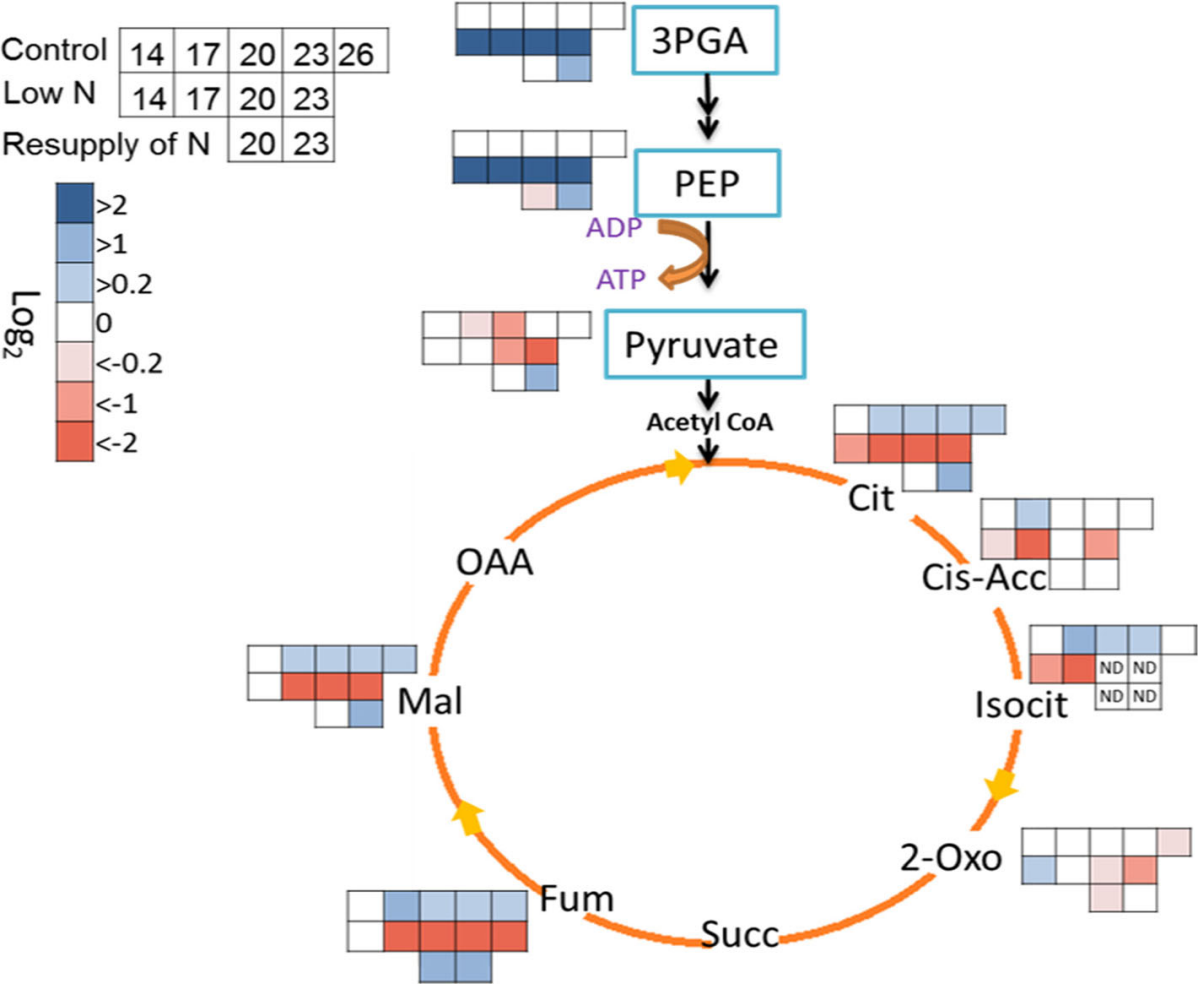
N-dependent changes in glycolysis and TCA cycle metabolites. Metabolite concentrations in control plants were logarithmic (log_2_) normalized to the values of control plants at 14 DAG; Results of low-N plants were logarithmic (log_2_) normalized to those of control plants at the same time point, while values of N resupplied plants were logarithmic (log_2_) normalized to those of low-N plants at the same time point. (*n* = 5–6); *P* ≤ 0.05. ND: not detected, 3PGA: 3-phosphoglycerate, PEP: phosphoenolpyruvate, Cit: citrate, Cis-Acc: cis-aconitate, Isocit: isocitrate, 2-Oxo: 2-oxoglutarate, Succ: succinate, Fum: furmarte, Mal: malate, OAA: oxaloacetate

Analysis of the metabolic rearrangement at the link between glycolysis and TCA cycle revealed an interesting N-sensitive response (Fig. 5). Two glycolytic intermediates, 3-phosphoglycerate (3PGA) and phosphoenolpyruvate (PEP), strongly accumulated in N-deficient plants (Fig. 5). Relative to control plants, their concentrations increased by 34- and 150- fold, respectively, after 6 d of N deficiency treatment (at 14 DAG). On the other hand, the concentration of pyruvate significantly dropped during growth under N deficiency (Fig. 5). Furthermore, pyruvate concentration did not yet change after 2 d of N resupply (at 20 DAG) but recovered to concentrations in control plants of the same age after 5 d of N resupply (at 23 DAG) (Fig. 5). The concentration of most measured metabolites of the TCA cycle slightly increased during growth under control conditions (Fig. 5). However, N deficiency caused a prominent decrease in all measured metabolites of the TCA cycle, and their levels mostly increased again when N was resupplied (Fig. 5).

**Fig. 6:**
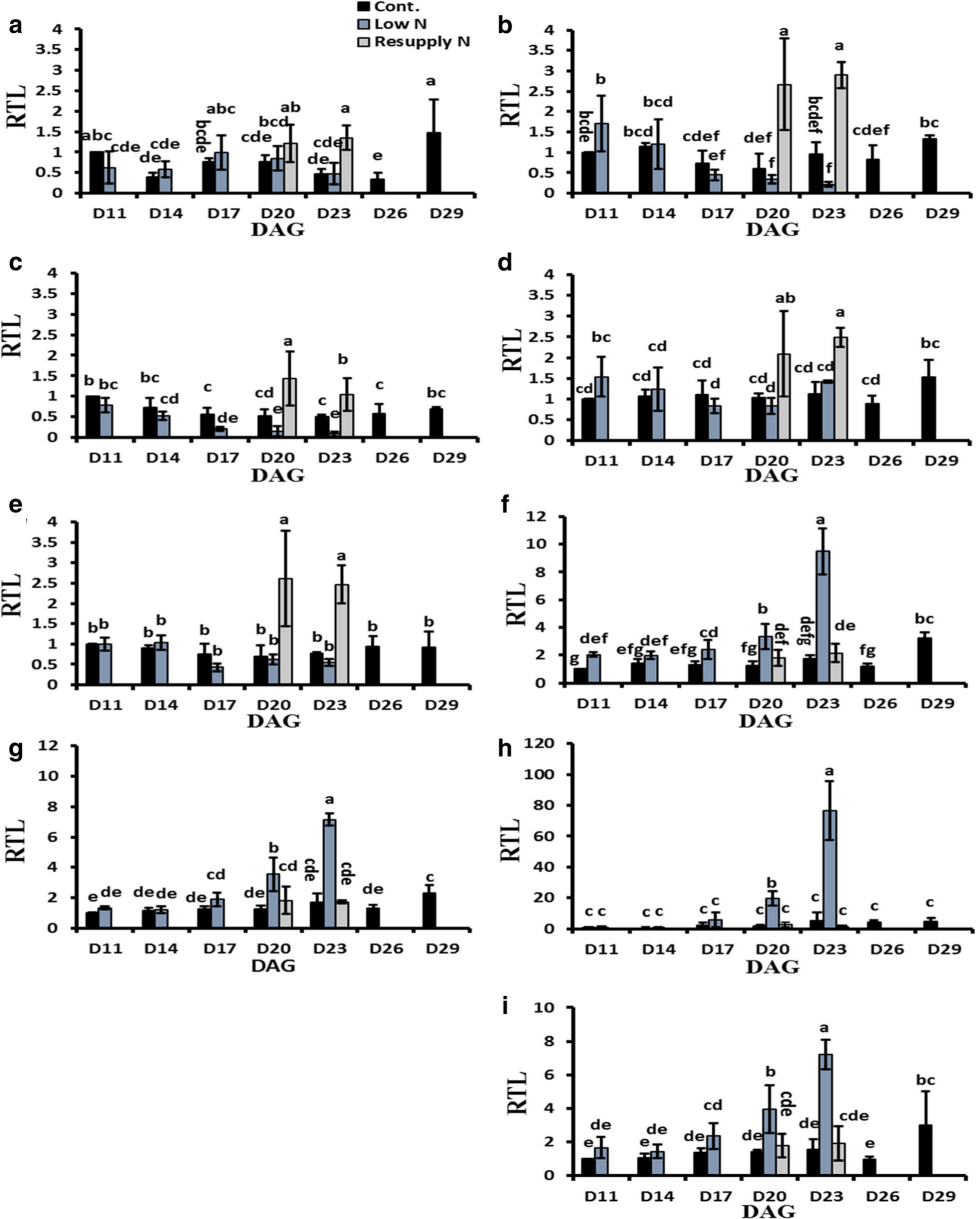
Transcriptional changes of genes involved in pyruvate metabolism under different N regimes. Transcript levels of **(a)** *PK1*; **(b)** *PK2*; **(c)** *PDH1*; **(d)** *PDH2*; **(e)** *PDH4*; **(f)** *PPDK1*; **(g)** *PPDK2*; **(h)** *PPDK3*; and **(i)** *PPDK4* in response to N deficiency and N resupply. Relative transcript levels (RTL) of all treatments were compared to those of control plants at 11 DAG. PK: Pyruvate kinase, PDH: pyruvate dehydrogenase, and PPDK: pyruvate, orthophosphate dikinase, DAG: day after germination, Bars indicate means ± SD (*n* ≥ 3). Different letters represent significant differences according to two-way ANOVA at *p* ≤ 0.05

The above-described metabolite profiling approach indicated that under N deficiency the flux of metabolites from glycolysis to the TCA cycle was disrupted at the level of pyruvate, suggesting that this step is a check-point for N-responsive regulation of C and N metabolism in barley. The transcriptome data also showed clear N-responsive regulation of genes related to glycolysis and TCA (listed in Table 2). Genes encoding pyruvate kinase (PK), pyruvate dehydrogenase (PDH), and pyruvate orthophosphate dikinase (PPDK) were differentially regulated in response to N-deficiency and/or N resupply (Table 2). To validate the microarray data and to investigate the changes in transcription of these genes in a more detailed time-scale, transcript levels were also analyzed by qPCR (Fig. 6). The results showed a distinct response pattern with a decrease in the mRNA levels of *PK2* and *PDH1* under low N, and a significant increase in *PK1*, *PK2*, *PDH1*, *PDH2*, and *PDH4* in response to N resupply to N-starved plants (Fig. 6). In contrast, all analyzed PPDK forms showed an opposite response with up-regulation under N deficiency and down-regulation after N resupply (Fig. 6).

**Table 2:** Differentially regulated genes putatively related to glycolysis and TCA cycle as determined via microarray analysis; the values represent fold change. Up arrow: Upregulated; down arrow: downregulated. Ct: Control, LN: Low N, RN: resupply N

ID	Putative coding	Low N (17 DAG)	Low N (20 DAG)	Resupply o (20 DAG)
hv_13593	Glyceraldehyde 3-P DH	↓2.3	↓3.2	↑3.2
hv_40636	Glyceraldehyde 3-P DH C subunit	↓9.9	↓6.4	↑5.4
hv_21406	Phosphofruktokinase	3.7	–	↓3.9
hv_09087	Phosphofruktokinase	–	↑2.7	↓2.2
hv_09091	Phosphofruktokinase	–	–	↓2.1
hv_39004	Plastidic pyruvate kinase beta subunit	↓2.8	–	–
hv_08013	Phosphoglycerate/bisphosphoglycerate mutase	2.5	–	–
hv_23968	Phosphopyruvate hydratase	–	↓2.3	↑2.9
hv_42359	Glucose phosphomutase	–	↓2.7	–
hv_18314	Enolase	–	–	↑2.1
hv_23969	Enolase	–	–	↑2
hv_21109	Pyruvate kinase 1 (PK1)	–	–	↑2.1
hv_40111	Pyruvate kinase 2 (PK2)	–	–	↑2.3
hv_11270	Pyruvate DH E1 subunit 1 (PDH1)	–	↓3.2	↑4
hv_16354	Pyruvate DH 2 (PDH2)	–	↓2.1	↑2.1
hv_16402	Pyruvate DH 3 (PDH3)	–	↓2.3	↑2.5
hv_41416	Pyruvate DH 4 (PDH4)	–	–	↑2.8
hv_36512	Isocitrate dehydrogenas	↓2.1		↑2.6
hv_10029	Malate DH	–	↓2.7	↑2.9
hv_41621	Malate DH	–	↓2	–
hv_41416	Dihydrolipoyllysine-residue acetyltransferas	–	–	↑2.8
hv_42774	Citrate hydro-lyase/aconitase	–	–	↓2.7
hv_00102	Pyruvate orthophosphate dikinase 1 (PPDK1)	–	–	↓4
hv_00103	Pyruvate orthophosphate dikinase 2 (PPDK2)	–	–	↓3.0
hv_22459	Pyruvate orthophosphate dikinase 3 (PPDK3)	↑4.9	↑5.4	↓7.2
hv_43181	Pyruvate orthophosphate dikinase 4 (PPDK4)	↑10.8	↑10.8	↓23.3
hv_19873	Malate synthase	–	↑3.1	–
hv_17622	Citrate synthase	–	–	↓3.3
hv_36529	Carboxyvinyl-carboxyphosphonate phosphorylmutase	–	–	2.4

## Discussion

Plant development strongly depends on uptake and transformation of N-containing compounds which build the biochemical basis of different metabolic functions. However, the availability of N is often limited, and frequently plants have to cope with periods of N shortage. Since N metabolism, together with C metabolism, is central to plant performance, the response of plants to low-N conditions affects many cellular functions, including N-responsive reprogramming of gene expression, synthesis/degradation of proteins and functional activation/deactivation of enzymes, which finally results in adjustment of metabolic pathways. In order to get insight into the regulation of primary N and C metabolism in response to changes in N availability, we analyzed both levels, the regulatory level of transcription and the metabolic response, via transcriptome and metabolome analyses under N shortage and N resupply after N shortage. As recorded by changes in relative chlorophyll content, root-to-shoot ratios and mRNA levels of chloroplast *GS2*, barley plants sensitively responded to decreased N availability and to resupply of N after N shortage (Fig. 1a, b, d). Moreover, also transcript levels of *S40*, a typical senescence marker, increased under N deficiency [19] (Fig. 1c), indicating that the N deficiency response resembles in part transcriptional changes during developmental leaf senescence. Thus, the present experimental set-up was suitable to identify metabolic processes playing a key role in the adaptation of plants to changes in N supply.

The array analyses revealed that barley leaves sensitively responded to changes in N availability by tremendous changes in transcript levels. N deprivation caused a major shift in the transcriptome by altering mRNA levels of about 2000 genes. Functional classification of these N-responsive genes indicated changes in N metabolism (protein, amino acids), transport processes, primary metabolism (photosynthesis, glycolysis, and TCA cycle), calcium signaling, redox regulation, hormone response and transcriptional regulation (chromatin structure, RNA, transcription and processing, and transcription factors), reflecting an adaptation of the leaf metabolic status to N supply. Previous investigations on N-deficiency responses in *Arabidopsis thaliana* and maize [12, 13] and on N resupply [11] reported similar changes in functional gene categories. Interestingly, about 62% of the genes that are regulated by N starvation showed a reversed regulation upon resupply of N to N-starved plants, indicating that plants sense N availability and adapt to increasing N availability by reverting a major part of the N deficiency response. In barley leaves, a major part of the genes responding to N availability was related to photosynthesis. These decreased under N deficiency and strongly increased again when N was resupplied. Other groups of genes were related to amino acid and protein degradation, as well as to amino acid and peptide transport, which represent central pathways during senescence to re-translocate N to sink organs (Fig. 2). In addition, several transcripts involved in lipid degradation were up-regulated in response to N deficiency, possibly to convert products from fatty acid breakdown via malate/pyruvate into carbon skeletons [21]. At the regulatory level, transcripts encoding NAC, MYB, MYB-related, Apetala 2/Ethylene-responsive element binding protein (AP2/EREBP), and Nodule Inception (NIN)-like transcription factors were up-regulated under N deficiency. These transcription factor families, except NIN-like, were also reported to be induced during developmental senescence in barley [22], indicating common regulatory pathways in the adaptation to N deficiency and in leaf senescence. On the other hand, transcripts encoding ARR-B, and C2C2-GATA were upregulated in response to resupply of N. Response regulators (ARRs) are playing a role in cytokinin signaling [23]. It was reported that *gnc* (GATA, nitrate-inducible, carbon metabolism-involved) mutants in Arabidopsis are more sensitive to exogenous glucose. Bi et al. [24] suggested a function of GNC in regulating carbon and nitrogen metabolism. Furthermore, a group of transcripts involved in redox regulation was down-regulated in N-deficient plants (Fig. 2), probably representing the loss of defense responses of N-starved leaves against oxidative stress. Changes in gene expression often are the first step in a series of events which finally ends in metabolic rearrangements. To better understand the program which changes leaf cellular functions in an N-responsive way, we investigated in addition to transcription, also alterations in the levels of N and C metabolites. Though changes in transcript levels do not necessarily result in gain- or loss-of-function of the encoded protein, we could identify by this approach several N responses that indicated transcriptional regulation of metabolic reprogramming.

Our metabolic analyses revealed that N starvation results in a substantial decrease in total amino acid concentrations of primary leaves, which could be reverted after resupplying of N (Fig. 3). As shown earlier [25], transcript levels of nitrate reductase sensitively responded to N availability, being down-regulated at low N and up-regulated at high N. Also mRNA levels of glutamate synthase were up-regulated after resupplying with N, while those of glutamate dehydrogenase were down-regulated. This opposite regulation in barley leaves in response to N availability reflects a metabolic shift, by which N assimilation via glutamate synthase becomes substituted by deamination of glutamate by glutamate dehydrogenase when plants are subjected to N deficiency. A similar N-sensitive shift in primary N assimilation has been described in several plant species [26, 27]. Moreover, transcript encoding glutamine synthetase 1 (*GS1*) was upregulated under N deficiency. Both, C and N metabolisms are closely connected and their interplay is supposed to play a role in senescence regulation [28, 29]. It has been reported that steam girdling of barley leaves leads to carbohydrate accumulation, especially of fructose, glucose, and sucrose, and that this carbohydrate accumulation could induce leaf senescence [30]. A strong connection between carbohydrate accumulation and the onset of senescence has already been reported before [31–33]. Here, we show that during N starvation, sugar metabolism undergoes a shift to producing more transport forms (sucrose), on the cost of starch (Fig. 4). Interestingly, trehalose-6-P (T6P) highly accumulated in N-deficient plants, which was reverted after N resupply (Fig. 4). Recently, trehalose-6-phosphate has been shown to regulate plant development, acting as a global regulator of metabolism and gene regulation [34, 35]. Our results support the idea that during the low N-induced onset of senescence T6P acts as a signal for high carbohydrate availability in plant cells to induce the senescence program. This is in agreement with a previous study showing that T6P content was altered by expressing the bacterial T6P phosphatase gene (*otsB*) to decrease T6P. *otsB-*expressing plants showed reduced anthocyanin accumulation and delayed senescence [36]. Our array analyses indicate that the specific metabolic shifts during N starvation and N resupply are related to the transcriptional regulation of genes involved in sugar metabolism. Many genes associated with sugar metabolism in barley leaves were affected by low N or by N resupply. Strong effects were observed for sucrose synthase (hv_03530 and hv_03531) and less strong effects for invertases, both being up-regulated under low N and down-regulated after N resupply, correlating with the observed accumulation of fructose (Fig. 4 and Table 1). These results oppose the notion that extracellular invertase activity supports the delay of leaf senescence via cytokinins [37]. Many other barley genes, putatively encoding fructokinase (hv_23265), two potential T6P phosphatases (hv_12166 and hv_10051) and starch degradation enzymes, which all are involved in sugar catabolism, showed an opposite regulation (Table 1). Interestingly, T6P synthase (hv_12166) and two putative T6P synthase/T6P phosphatase genes (hv_04008 and hv_04009) were also down-regulated during low N and up-regulated after resupplying of N (Table 1). This is opposite to the observed strong accumulation of T6P at low N and might reflect a feed-back mechanism. However, the exact functions of the proteins encoded by the two putative T6P synthase/ T6P phosphatase genes from barley are needed to be clarified in future.

Our transcriptomic and metabolic analyses further suggest that N deficiency results in a major reorganization of primary C/N metabolism. Notably, PEP and 3PGA are highly accumulated upon N deficiency, while pyruvate levels decreased (Fig. 5). This finding hasn’t been reported in other plant species, such as maize or *Arabidopsis thaliana* [11, 12]. In addition, we report here a decline in the majority of TCA cycle components upon N deficiency (Fig. 5), which is in contrast to a report of Comadira et al. [13] and might go back to differences in the experimental design. Organic acids form the skeleton for amino acids, however, there was no clear correlation in the patterns of the different organic acids and their related amino acids (Fig. 3b and 5). Our metabolic analyses demonstrate that the pyruvate biosynthesis step controlling the metabolite flow from glycolysis to the TCA cycle is a crucial step and sensitively reacts to N availability. These changes are reflected by a specific regulation at the transcriptional level. While genes encoding pyruvate kinases (1 and 2) were not regulated in response to N deficiency and only slightly up-regulated after resupply of N, transcript levels of the enzymes catalyzing the reverse-reaction from pyruvate to phosphoenolpyruvate, which is pyruvate orthophosphate dikinase, were strongly up-regulated during the low-N treatment and strongly down-regulated after N resupply (Table 2 and Fig. 6). Senescence-specific up-regulation of pyruvate orthophosphate dikinase in Arabidopsis leaves has already been described. The authors discussed that the accumulated phosphoenolpyruvate is finally transformed to the transport form glutamate to ensure N export to other parts of the plant [38]. However, in contrast to the report by Taylor et al. [38], we did not observe an accumulation of the intermediates citrate, isocitrate, and 2-oxoglutarate (Fig. 5), which in the present experiment might have been transformed at high rates directly to form glutamate. In fact, despite a general decrease in glutamate during N starvation, its proportion relative to the total amino acid concentration clearly increased during N starvation. Thus, the present data suggest that by induction of the reverse reaction back to phosphoenolpyruvate during N starvation an efficient pathway for remobilization of N sources is established and that this is at least partly regulated by a strict reprogramming of the transcript levels of pyruvate orthophosphate dikinase.

## Conclusion

Barley plants respond to changes in N supply at different cellular levels, which includes as an early regulatory event major reprogramming of gene expression and ends in the sophisticated rearrangement of metabolic pathways. Decreasing N availability affects the expression of more than 2000 genes and most of them belong to major functional classes including photosynthesis, cell wall degradation, lipid degradation, amino acid degradation, hormonal signaling, and regulatory factors, e.g. transcription factors**.** Interestingly, 62% of these genes were regulated in the opposite direction after resupplying of N, indicating a strong signaling pathway of N-responsive gene expression. This strong reprogramming of gene expression results in major metabolic rearrangements of cellular functions, mainly in N and C metabolism. Besides major shifts in sugar and amino acids metabolism, we show a sensitive reaction of pyruvate metabolism at the link between glycolysis and TCA cycle, which interestingly involves N-sensitive transcriptional regulation of pyruvate orthophosphate dikinase, encoding the enzyme responsible for the reverse reaction from pyruvate to phosphoenolpyruvate. These results indicate induction of a reverse reaction from pyruvate back to phosphoenolpyruvate during N starvation as an efficient pathway for the remobilization of N sources.

## Additional files

Additional file 1: **Table S1.** Primers sequence that were used for qPCR analysis (PDF 404 kb)

Additional file 2: **Table S2.** List of the all genes regulated by N status. You can find the table in the attached excel file. The relative expression was determined by microarray. The values are presented log_2_ (fold changes (FC)). Values of Low N are relative to corresponding time point of control. Whereas Values of N resupply are relative to Low N (20 DAG). (XLSX 634 kb)
