# Supplementary material for: Changes in nitrogen availability lead to a reprogramming of pyruvate metabolism
Source: BMC Plant Biol. 2018 May 4;18:77. doi: 10.1186/s12870-018-1301-x (PMC5935972; doi:10.1186/s12870-018-1301-x)
Supplement: Supplementary file 1 — Table S1. Primers sequence that were used for qPCR analysis (PDF 404 kb) [file 12870_2018_1301_MOESM1_ESM.pdf]

Supplementary Materials:

Table S1: Primers sequence that were used for qPCR analysis

Gene	Forward primer	Reverse primer
Hv-S40	GTCTGCCCCGGTCCTCGTG	GTTCCTCTTCGCGTCGTTGG
Hv-PP2A	CACCATTTCTCAGCTTGTATTG	CACCCCTTTGTTATTGTTTGTTG
Hv-GS2	AGCGCGATCTCACAGGTCG	ATCGTCGTCTCTACGTACTTGC
Hv-actin	GGAAATGGCTGACGGTGAGGAC	GGCGACCAACTATGCTAGGGAAAAC
HvPK1	CTTTTCCTGCATCTCCCCTGA	TGGTCACAGGCAAGCACATA
HvPK2	CGTGTAATTAGTGGTTTTCGTCGTC	GGCCAACAAACAACAGCTAATGATA
HvPDH1	CATCGGGAACTCCATCAAGAAGA	AGTGTCCAGCTCATCCCAGAAG
HvPDH2	CGACAAAACACAAAGGCGATAAAC	GAGGAGGAGATGGATGGAGAGGAC
HvPDH4	CATCGTCCTCCTCGGCTAATCC	GGTTCCCTTGCTGTTGTTGTTG
HvPPdK1	CACTTTCCCCACCAGCCCTTAG	ATTCCCTCTGCCCCATTCTTCC
HvPPdK2	CACGCAGATGACATTTGGTTACA	CAAAGGGGTCATGCTGGAGGATA
HvPPdK3	TTGCACTCACTAATGGCTCC	GTCTTTCATGGCCTTGTTGC
HvPPdK4	AGAACGACACCGACCTGACTG	CTCCTTTGGCTTCGACATAGAC
